# Oral Health Outcomes In An HIV Cohort With Comorbidities- Implementation Roadmap For A Longitudinal Prospective Observational Study

**DOI:** 10.21203/rs.3.rs-3390162/v1

**Published:** 2023-10-04

**Authors:** Temitope Omolehinwa, Sunday O. Akintoye, Marta Gabinskiy, Vincent Lo Re, Mel Mupparapu, Rosa Urbina, Douglas E. Schaubel, Patricia Corby

**Affiliations:** University of Pennsylvania; University of Pennsylvania; University of Pennsylvania; University of Pennsylvania; University of Pennsylvania; University of Pennsylvania; University of Pennsylvania; University of Pennsylvania

**Keywords:** HIV, non-infectious comorbid conditions, oral health, dental caries, periodontal disease, inflammation

## Abstract

Long-term antiretroviral therapy (ART) perpetually suppresses HIV load and has dramatically altered the prognosis of HIV infection, such that HIV is now regarded as a chronic disease. Side effects of ART in Patients With HIV (PWH), has introduced new challenges including “metabolic” (systemic) and oral complications. Furthermore, inflammation persists despite great viral load suppression and normal levels of CD4^+^ cell count.

The impact of ART on the spectrum of oral diseases among PWH is often overlooked relative to other systemic complications. There is paucity of data on oral complications associated with ART use in PWH. This is in part due to limited prospective longitudinal studies designed to better understand the range of oral abnormalities observed in PWH on ART. Our group designed and implemented a prospective observational longitudinal study to address this gap.

We present a procedural roadmap that could be modelled to assess the extent and progression of oral diseases associated with ART in PWH. We described here the processes associated with subject recruitment and retention, study visit planning, oral health assessments, bio-specimen collection and preprocessing procedures, and data management. We also highlighted the rigors and challenges associated with participant recruitment and retention.

## Background

HIV infection was initially associated with high mortality, but the discovery of ART and most recently long-acting injectable ART (1), has significantly reduced morbidity and mortality PWH. HIV has become a chronic disease, and life expectancy among PWH is approaching that of HIV-uninfected persons. HIV disease is still a global health challenge, affecting about 39 million people worldwide (2)

ART reduces AIDS-associated morbidity and mortality by increasing peripheral recovery of the CD4^+^ T-cell compartment and reducing viral load(3), but PWH still experience decreased metabolic control and increased rates of metabolic diseases as side effects of ART. These metabolic diseases are believed to affect up to 50% of PWH (4). This includes effects on lipid and glucose metabolism, with patients at higher risk for developing dyslipidemia (80% prevalence), lipodystrophy, insulin resistance, diabetes (with protease inhibitors as the major culprit)(5) and other cardiovascular diseases (CVD) (6, 7, 8, 9). PWH on ART also have chronic/persistent activation and inflammation of the immune system, due to disruption of regular activities of cytokines, coagulation factors, chemokines, microbial translocation especially noted in the gut, and T effector cells(3, 10, 11). This is caused by persistent low-level viral replication extracellularly, as ART prevents the virus from entering the CD4^+^ cells. Immune activation is further heightened by factors such as co-morbid conditions especially metabolic syndrome associated with hypertension, hyperlipidemia, insulin resistance and obesity; co-infections with herpes simplex virus, tuberculosis; possible premature or abnormal slowing down of the immune system; and tobacco smoking(3, 6, 12). Monitoring immune activation in addition to CD4^+^ cell count is clinically useful in patients on long-term ART (13). The range of metabolic abnormalities observed in PWH on ART suggests that these metabolic abnormalities play a critical role in supporting and driving chronic immune activation and inflammation in HIV infection.

The oral cavity is not spared from deleterious side effects of ART, due to HIV-related changes(14). Long-term suppression of HIV also seems to be accompanied by excessive deleterious inflammation that could promote oral complications such as periodontitis. This is due to periodontitis promoting activation of systemic inflammation accompanied by viral replication in PWH(15, 16). Periodontitis is an oral disease, and a contributor to the global challenge of chronic diseases(17). Although severe oral opportunistic infections decreased with the implementation of ART, periodontitis is still a commonly described disease in PWH.

The direct role of immune activation or chronic inflammation on salivary gland dysfunction in PWH is unclear, but it is well established that HIV infection affects the salivary glands. Moreover, ART is also associated with a high risk of xerostomia, especially in patients on protease inhibitors(18).

Despite generally reported decreases in oral complications post ART(19), there is still an increase in poor oral health conditions in PWH. Shiboski et al in 2018 reported that 86% of youths living with HIV on protease inhibitors developed dental caries, while ART-naïve participants had a lower caries rate (20).

Additionally, the interplay between the immune and skeletal systems, often described as the “immunoskeletal interface” has been implicated in the pathogenesis of bone diseases in the setting of HIV infection(21), with high rates of osteopenia and osteoporosis reported among PWH(22). Lymphocytes have been implicated in receptor activator of nuclear factor kappa-B ligand (RANKL) production and bone loss associated with skeletal pathologies that include postmenopausal osteoporosis(23), inflammatory rheumatoid arthritis(24) and alveolar bone loss associated with periodontal infection(25). Initiation of ART has been consistently associated with up to a 6% reduction in hip bone mineral density (BMD). However, some ART regimens may be associated with more pronounced bone loss(26, 27, 28, 29), in patients at risk for fractures especially vertebral fractures(27). Dual-energy x-ray absorptiometry (DEXA) is the standard of care for evaluating BMD changes, but there is limited information on whether panoramic radiographs (PAN) can be used as a tool to assess BMD changes in PWH (27, 30, 31).

The impact of oral health on systemic health is an understudied area, especially in HIV research. There remains a wide gap in our understanding of the interplay of poor oral and systemic outcomes in PWH. Our group developed and designed a prospective longitudinal study, with a goal to prospectively investigate the range of metabolic abnormalities observed in PWH on ART. We present the design details of our longitudinal observational clinical study as it may serve as a roadmap for other studies testing the extent and progression of oral diseases associated with ART in PWH. It also described the challenges of a study launched during the Covid-19 pandemic and how some of these challenges were navigated.

## Study Design and Methods

### Study Hypothesis

**Figure 1:**
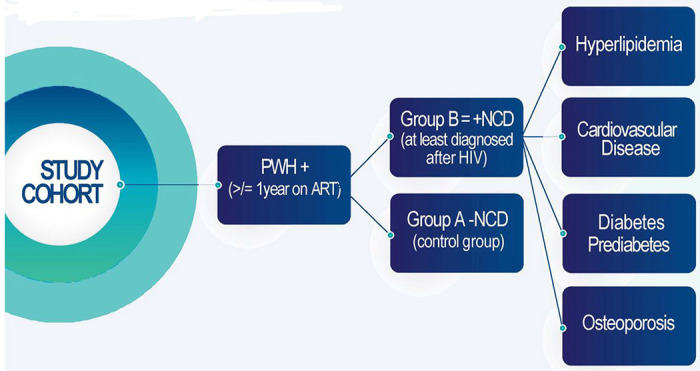
Study Cohort: Patients with HIV on ART (≥1 year). Group A: Participants with no comorbidities. Group B: Participants with ≥1 comorbid disease diagnosed after ART initiation.

The central hypothesis was that the extent and progression of key oral diseases (e.g., caries, periodontal disease, and oral cavity confections) are associated with ART-induced salivary changes and exacerbated in PWH who have been on ART for at least one year and have developed non-communicable diseases (+NCDs) including diabetes, cardiovascular diseases, osteoporosis, and hyperlipidemia after ART initiation. PWH without NCDs (−NCDs) served as control group (Figure 1). The secondary hypothesis was that patients in the +NCD group will present with higher degrees of salivary gland hypofunction (e.g., dry mouth); greater changes in levels of salivary proteins, as well as higher levels of salivary inflammatory and immune activation cytokines. We further included an exploratory hypothesis, that BMD changes noted on panoramic radiographs are predictors of osteoporosis risk when compared to the gold-standard DEXA scans.

### Subject Recruitment, Timeline and Inclusion and Exclusion Criteria:

Target enrollment was 350, with a study duration of 24 months (2 years). Study visits and data collection were scheduled every 6 months for PWH above the age of 18 years, who have been on ART drugs for at least 12 months. Participants with xerostomia-related autoimmune conditions like Sjogren’s, and sarcoidosis; head and neck radiation therapy (including radioactive iodine therapy); chemotherapy within the prior year before enrollment in the study; use of anti-osteoporotic agents (Bisphosphonates, Denosumab); and pregnancy at time of enrollment, were excluded.

Participants were categorized into two groups of +NCD and −NCD patients as described in Figure 1.

Eligibility was confirmed by review of medical/dental records of patients with a diagnosis of HIV and eligible subjects were contacted by telephone or approached during clinical care. Written informed consent was obtained at the baseline study visit for interested participants, after thorough review of all study visit procedures in layman terms and answering questions from participants. The study was carefully designed to include only PWH. Also, PWH who have not commenced ART drugs and HIV-uninfected individuals were excluded, because the study focused on evaluating the effect of ART on oral and systemic health outcomes. This approach allowed for assessment of onset (when possible) and progression of oral and systemic diseases.

Participants who have been on ART for less than one year were also excluded because it was necessary to capture onset and progression of ART related non-communicable conditions. The rationale for the timeline was based on current research suggesting that most NCDs are diagnosed approximately 12-24 months after ART initiation(32).

Subjects were followed-up for 2 years to compare findings on panoramic radiographs (PAN) with those on DEXA scans, especially findings that related to changes in BMD. The goal was to ultimately determine if PAN is a sensitive tool for an early diagnosis of bone disease among PWH in a dental setting.

### Study Procedures

#### Clinical Data Collection

The following demographic data were collected: age, biological sex, gender identity, sexual preferences, ethnicity, and race. Medical history captured included past and current medical conditions, and currently prescribed and over-the-counter medications.

#### Medical and Oral health Questionnaire

Oral health, xerostomia-related quality of life (XeQoLs)(33, 34) using the National Health and Nutrition Examination Survey (NHANES) 2012 (35) and modified XeQoLS questionnaire (36) respectively; and Social history using the Tobacco, Alcohol, Prescription medication, and other Substance use (TAPS) tool (https://cde.nida.nih.gov/instrument).

#### Standard of Care (SOC) Clinical Labs

CD4^+^ cell count, viral load, and lipid panel (including HDL, LDL, and total cholesterol levels) were abstracted from medical records. Point of care glucose levels were assessed chair side.

#### Vital signs and Physical measurements

Vital signs obtained included weight, temperature, blood pressure and heart rate. Height was measured at baseline only.

#### Stimulated Saliva Collection

Stimulated saliva was preferably obtained in the morning due to the circadian pattern of saliva production. Participants were given a 1.25 x 0.25 inch Parafilm cube from a pre-weighed 50mL conical tube and instructed to chew continuously and avoid swallowing for 5 minutes. An autoclaved funnel was placed inside the conical tube, with the tube embedded in wet ice. At the end of each minute, participants were asked to gently spit pooled saliva into the funnel. After 5 minutes, the remaining saliva and parafilm were expectorated into the funnel. The tube was transferred to the laboratory for preprocessing in a cool box.

Saliva flow rate was determined based on comparing pre and post saliva collection weights of the conical tube. The saliva was also centrifuged at 4°C, 2,500 rpm for 25 minutes to separate supernatant and pellet. Pierce Protease Inhibitor cocktail EDTA-free was added at per 1uL/100uL of saliva supernatant for up to 2mL of saliva. Saliva pellet was also transferred into a cryovial tube containing 500ul of DNA/RNA Shield^®^ Solution. Saliva supernatant with and without protease inhibitor and the saliva pellet were stored at −80°C. Expression levels of inflammatory and immune activation cytokines in saliva supernatant were measured using Polymerase chain reaction (PCR), while pellets retrieved were stored for future microbiome and genome studies.

#### Blood sample collection

Patients were asked to fast for approximately 8 hours prior to collection of blood samples. Patients with diabetes, especially those on insulin, were prioritized for the first visit of the day.

A glucometer was used to assess point of care fasting blood glucose. In addition, approximately 22mL of blood was collected by venipuncture in the following order: A one-time collection of 2 PAXgene^™^ blood RNA tubes for future genomics studies. This was preceded by collecting approximately 1mL of blood in a discard tube; one EDTA tube (purple top) for collection of plasma samples for analysis of inflammatory and immune activation markers; and two Serum separator tubes (tiger top) for collection of serum sample for analysis of inflammatory and immune activation markers, and the other for assessment of total insulin levels.

The plasma samples were preprocessed within 30 minute of sample collection, while one of the two tubes containing serum was preprocessed after 30minutes. Samples were stored at −80°C. Expression levels of inflammatory and immune activation cytokines were assessed by PCR.

PAXgene^™^ tubes were stored at room temperature for 2 hours, transferred to −20°C freezer for 72 hours and up to 30 days before long term storage at −80°C for future genomic studies.

#### Oral Health Assessment

Teeth with decayed, missing and filled surfaces (DMFS) were recorded, and DMFS index was used to determine participants’ caries experience. While decayed teeth show the presence of active caries in the mouth, missing teeth could imply tooth loss due to periodontal disease, from non-restorable caries or other factors.

Periodontal probing depth, clinical attachment loss and bleeding indices were also assessed to evaluate periodontal health status. The American Dental Association and American College of Cardiology guidelines was followed regarding antibiotic prophylaxis for participants (37).

An intraoral soft tissue assessment was performed by calibrated examiners to determine the presence or absence of oral mucosal lesions, such as candidiasis, red/white lesions, warts, and ulcers. The oral cavity was also examined for xerostomia by objective assessment of dry oral cavity, stickiness of dental mirror to the oral mucosa, and reduced or loss of saliva flow from the Stenson and Warton ducts bilaterally. All dental examinations were performed according to SOC guidelines.

#### DEXA Scan

Whole body DEXA measurement, which includes femoral neck (hip) and lower spine, was performed using the Horizon A Platform (Hologic Inc.; Bedford, MA) with Apex software v5.5, to estimate BMD. T and Z-scores were measured using the NHANES database SD(38).

#### Panoramic Radiographs

Panoramic radiographs (Planmeca Promax^®^ 2D S3 series, Promeca Corporation, Helsinki Finland) were taken to identify overall image radiographic density as well as changes associated with osteopenia. Such changes include thinning of the mandibular cortical bone (lower border of the mandible) and presence or absence of the trabecular bone. The goal was to compare panoramic radiographic data with T and Z-scores of DEXA scans with respect to risk for osteoporosis and/or osteopenia.

#### Oral Swabs

Swabs were taken from the dorsum of the tongue and any other intraoral site with suspected candidiasis, using the Isohelix^™^ buccal swabs. The cotton end of the swab stick was then placed in a 2mL tube containing DNA/RNA shield^®^. Samples were vortexed for 30 seconds and stored at −80°C, for future quantitative analysis using PCR to detect the presence of candida species.

#### Urine pregnancy test

Urine pregnancy test was performed on women of childbearing potential prior to each imaging study, unless they were over the age of 45 and had not had a menstrual period for at least 12 months prior to enrollment. If female participants were noted to be pregnant at the follow up visit which required imaging, they were allowed to continue with the study visit but imaging studies were excluded.

### Visit Schedule

**Figure 2:**
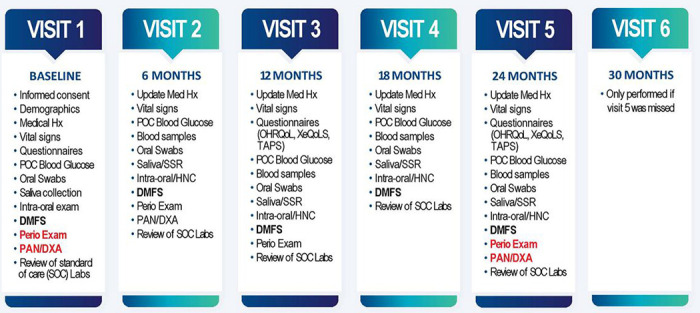
Visit Schedule: Shows all study procedures per study visit.

A schematic representation of the visit schedule is shown in Figure 2. SOC laboratory study reports were reviewed at every study visit. If results within 12 months of study visits were unavailable, new tests were requested from the patients’ primary care or infectious disease physician. Confirmation of HIV diagnosis was by SOC blood tests. Screening and baseline visits were usually performed on the same day.

Participants who chose to withdraw from the study were not required to attend an in-person withdrawal visit. The reason for withdrawal, if known, was documented in the source documents and study database. Protocol deviations including missed study visits were well documented in the protocol deviation form, and adverse events were assessed and appropriately documented.

### Data Capture

Data for our study were captured using Research Electronic Data Capture (REDCap version 13.8.0 developed by Vanderbilt University), an electronic data capture, management and reporting tool, under the management of the Clinical Research Computing Unit (CRCU), School of Biostatistics and Epidemiology University of Pennsylvania, with restricted access to authorized personnel and study team members only.

### Sample size and Statistical analysis

The power/sample size assessment was based on the primary analysis for comparing prevalence of periodontal diseases between the +NCD and −NCD groups. From the pilot sample, the probability of periodontal disease was 0.203 and 0.373 for the −NCD and +NCD groups respectively, and the estimated odds ratio (OR) for periodontal disease for −NCDs subjects relative to +NCDs subjects was OR=2.33 [(0.373/(1-0.373)/(0.203/(1-0.203)].

The required sample size was determined based on a primary analysis using logistic regression; a baseline probability of periodontal disease of 0.203 (for −NCD subjects); 50% exposure prevalence; and power of 80% using a significance level of 0.05. A sample size of 302 allows detection of an OR of 2.1 with 80% power. To accommodate an anticipated drop-out rate of approximately 10-12%, the target sample size was increased to n=350, to allow for a 13.7% dropout rate, while maintaining the afore-described power.

For descriptive analysis, comparison of continuous variables will be based on either t-tests or the Mann-Whitney test, while comparison of binary variables will use either the Chi- Square test or the Fisher’s exact test, depending on assumptions for each test. Primary statistical analysis will use Logistic regression to assess the odds ratio for the outcome for subjects in the +NCD group relative to subjects in the −NCD group. Data analysis will be performed with Stata SE v16 and SAS v9.4.

## Discussion

Large scale prospective longitudinal studies involve following subjects over an extended period of time and are only successful when the study design process is well thought out prior to starting the project. Key design considerations as are described below:

### Access to target patient cohort

Accessibility to target patient population in a prospective longitudinal study is important to meet the recruitment goals. Both Penn Dental Medicine (PDM) and the Center for AIDS Research (CFAR) at the University of Pennsylvania have access to a large cohort of PWH. The partnership with CFAR was critical for the success of our recruitment.

### Study team

**Figure 3:**
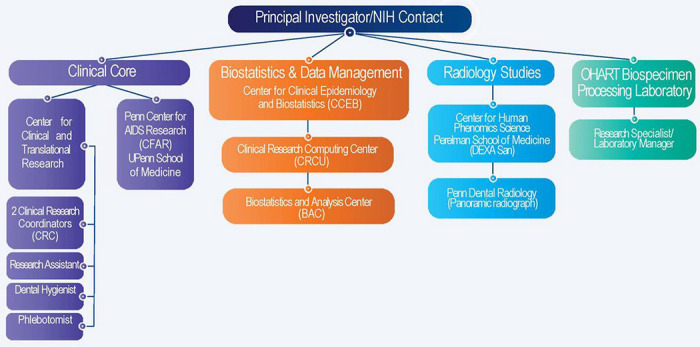
OHART Study Organogram: Shows the multidisciplinary team of collaborators involved in the study development and execution.

Project development in clinical and translational research works best with a multidisciplinary team of collaborators and experts including statisticians, domain experts, and data managers. In our case, oral health professionals, infectious disease experts, clinical and translational research core, data management team and biostatisticians met together to develop this project (Figure 3). This interdisciplinary collaboration of experts was assembled at the University of Pennsylvania, between investigators from Oral Medicine department and Clinical and Translational Research Center (CTRC) at PDM as well as experts at Penn School of Medicine (PSOM) CFAR.

The study team included one research dental hygienist, two clinical research coordinators, two research specialists (this included a laboratory manager), one research assistant, multiple dentists/oral medicine specialists, a biostatistician and one phlebotomist. Most team members were cross trained in different research tasks, for example several team members were cross trained on phlebotomy.

Calibration based on study procedures e.g., oral health assessment, sequence of study procedures, recording of adverse events, etc., was done on an annual basis.

### Funding

Adequate funding is an essential part of a clinical study. This project was funded by the National Institute of Health/National Institute of Dental and Craniofacial Research/Department of Health and Human Services (NIH/NIDCR/DDHS), with grant number # R01-DE029648.

### Institutional Infrastructure

A well-established infrastructure can enhance the quality of research, ensure patient safety, and increase the likelihood of obtaining meaningful results.

PDM CTRC is a core facility that provides support services for the development and conduct of clinical research. This includes support for protocol and manual of procedure development, grant submissions, ethical considerations, regulatory compliance, and data and quality management review. Our group worked closely with the PDM CTRC in the development and implementation of the study.

Potential challenges with planning and conducting a human subjects’ research include costs, inability to assemble a team of appropriate experts in the field of study as collaborators, lack of essential infrastructure, inability to meet enrollment targets, and challenges with retention.

Delays in recruitment can prolong study duration, resulting in increased costs and delayed results. A study that does not enroll the required number of participants may lack statistical power to detect a significant difference or effect, making the results inconclusive.

These challenges are common, especially for a study initiated during the Covid-19 pandemic. The first participant on our study was enrolled in February 2021, when people were still wary of visiting a dental healthcare facility.

Regular monitoring and adaptive strategies help in addressing enrollment challenges as they arise. This includes community engagements as well as other technologically innovative strategies. To overcome enrollment challenges, we partnered with the UPENN HIV community outreach and advocacy program. We also increased our engagement with UPENN CFAR.

Other challenges encountered during this study include loss-to-follow up of subjects and staff turnover. The workforce was significantly impacted, as staff hiring became difficult and staff members were sometimes uncomfortable with conducting studies during the Covid-19 pandemic, and some found the added layers of personal protective equipment somewhat daunting.

At the time of writing this article, approximately 24 months into the study, 157 out of 350 participants had been enrolled. 2.6% (n=4) withdrew from the study, 0.7% (n=1) was withdrawn by the principal investigator (PI) and 8.6% (n=13) were lost to follow up. Participants that missed two consecutive study visits were considered lost to follow-up. We were able to overcome the challenge of missed visits by reconfirming appointments a week before, a day before and when necessary, on day of appointment. Other challenges include patients sometimes presenting for their appointments without fasting, as well as inability to collect saliva samples in the morning on all participants due to patient related factors, like participant availability in the mornings.

A rigorous prospective longitudinal study requires proper planning and execution. A great benefit is that large data sets collected and biospecimen repository can be used to answer more questions in future studies including genetic, microbiome and metabolome-based studies. In addition to answering the research questions posed in this study, future directions include evaluating the role that genetics, environmental and social factors as well as oral microbiome play in the interplay of oral and systemic health in PWH. These new studies generate evidence for oral health treatment guidelines tailored to the needs of dental patients with HIV.

## Data Availability

Protocol and MOP for this study are both available on request from the first author. Data collection for this project is still ongoing and will be made available to the public as soon as possible

## Abbreviations

ART
CFAR: Center for AIDS Research
DHHS: Department of Health and Human Services
DEXA: Dual Energy X-ray Absorptiometry
DMFS: Decayed Missing and Filled Surfaces
DNA: Deoxyribonucleic Acid
EDTA: Ethylene Diamine Tetraacetic acid
HIV: Human Immunodeficiency Virus
ICF: Informed Consent Form
NCD: Non Communicable diseases
NHANES: National Health and Nutrition Examination Survey
NIDCR: National Institute of Dental and Craniofacial Research
NIH: National Institute of Health
PCR: Polymerase chain reaction
PAN: Panoramic radiograph
PDM: Penn Dental Medicine
PWH: Patients with HIV
REDCap: Research Electronic Data Capture
RNA: Ribonucleic Acid
SOC: Standard of Care
TAPS: Tobacco, Alcohol, Prescription medication, and other Substance use
UPENN: University of Pennsylvania
XeQoLs: Xerostomia-Related Quality of Life
